# Combined therapy with the estrogen receptor ERα and IFNα-2b suppresses HBV replication by inducing GBP1 expression

**DOI:** 10.3389/fimmu.2025.1698786

**Published:** 2026-01-16

**Authors:** Yadi Li, Jiaojiao Gong, Guili Tan, Haiying Luo, Xiaoxia Hu, Bo Qin

**Affiliations:** 1 Department of Gastroenterology and Hepatology, Laboratory for Clinical Medicine, Beijing You’an Hospital Affiliated with Capital Medical University, Beijing, China; 2 Department of Infectious Diseases, Chongqing Key Laboratory of Infectious Diseases and Parasitic Diseases, The First Affiliated Hospital of Chongqing Medical University, Chongqing, China; 3 Central Laboratory, The First Affiliated Hospital of Chongqing Medical, University, China

**Keywords:** Chronic hepatitis B, estrogen receptor, guanosine-binding protein 1 (GBP1), Hepatitis B virus, Pegylated interferon α-2b, Replication

## Abstract

Pegylated interferon α-2b (peg IFNα-2b) is a first-line clinical drug for the treatment of chronic hepatitis B (CHB). It can effectively reduce HBsAg levels, improve clinical cure rates, and lower the incidence of HBV-associated hepatocellular carcinoma (HCC). Notably, females consistently exhibit a better early response to interferon therapy than males do. Previous studies have confirmed that the estrogen receptor ERα inhibits HBV transcription. However, reports on combined treatment with ERα and peg IFNα-2b for HBV infection are limited. Hepatocyte RNA-seq analysis revealed that both ERα elevation and IFNα stimulation can lead to increased expression of GBP1. GBP1 has been reported to inhibit several bacteria and HCV, but evidence of its effect on HBV infection is insufficient.We systematically investigated the roles of ERα, peg IFNα-2b, and GBP1 in modulating HBV infection in an HBV-infected HepG2-NTCP cell model. Molecular dynamics simulation and molecular docking methods were employed to analyze the stability and interaction sites of the ERα and GBP1 complex. Finally, we examined the RNA and protein levels of GBP1 and ERα in the peripheral blood of CHB patients treated with peg IFNα-2b.GBP1 was induced by costimulation with ERα and peg IFNα-2b. The combination of ERα and peg IFNα-2b enhanced the inhibition of HBV replication, which might be achieved by promoting GBP1 expression and altering its cellular distribution. GBP1 was highly expressed in the PBMCs and liver tissues of CHB patients and was specifically positively correlated with ERα. Interestingly, GBP1 was enriched in the NOD-like signalling pathway, which activates the host immune response.Our study demonstrated that the combination of ERα and peg IFNα-2b exerts antiviral effects by mediating the high expression of GBP1. These findings reveal the associations among ERα, peg IFNα-2b, and innate antiviral immunity, which target GBP1.

## 1 Introduction

As a hepatotropic DNA virus, the hepatitis B virus (HBV) specifically infects hepatocytes by binding to the sodium taurocholate cotransporting polypeptide receptor (NTCP), progressing to acute or chronic hepatitis and significantly increasing the prevalence of cirrhosis and primary liver cancer (1, 2). Despite the global availability of a safe and effective HBV vaccine to prevent viral infections, 240 million people still develop chronic hepatitis B (CHB), which poses a serious threat to public health (3, 4). Additionally, the prevalence of CHB varies regionally. A markedly higher infection rate has been reported in China, India, and other densely populated regions (5). The ideal clinical endpoint for CHB treatment is to achieve a functional cure, wherein HBV DNA and HBsAg levels are undetectable after a fixed treatment course, with or without the production of HBsAb, thereby mitigating liver pathology and improving its biological functions (6, 7). However, HBV has evolved sophisticated immune evasion strategies that impair host immune surveillance and facilitate chronic infection. Notably, a novel T-cell subset (CD3^+^CD4^-^CD7^+^CD57^-^a) has been found to decline significantly in patients with HBV-related end-stage liver disease, suggesting that virus-induced immune remodeling may contribute to disease progression and suboptimal antiviral responses (8). In addition to its intrinsic pathogenicity, HBV often co-infects with other bloodborne viruses such as HIV and HCV due to shared transmission routes. These coinfections complicate clinical management and are frequently associated with more severe liver damage, higher rates of chronic infection, and reduced therapeutic efficacy. Furthermore, the immunopathogenesis of HBV can be exacerbated under coinfection conditions, as overlapping immune escape mechanisms and cytokine disturbances between viruses may synergistically intensify hepatic injury and disease progression (9). In the current state of research, nucleos(t)ide analogs (NAs) and interferon-alpha (IFN-α) are approved for the treatment of CHB. Although these two drug classes significantly suppress viral replication and delay hepatic pathological changes, they do not completely eradicate HBV (10). Compared with NAs, IFN-α has several advantages, including higher seroconversion rates for HBeAg and HBsAg, longer-lasting responses, and a lower incidence of drug resistance (11). IFN-α binds to surface receptors on target cells, stimulating the classical JAK-STAT signalling pathway to induce the expression of interferon-stimulated genes (ISGs), thereby modulating immune responses (12, 13), or it directly acts on various steps of HBV transcription and replication to exert antiviral effects (14). Furthermore, IFN-α enhances host innate immune mechanisms by producing anti-inflammatory cytokines and chemokines to counteract the immune dysregulation caused by chronic HBV infection (15). IFN-α is also capable of secreting cytokines that promote intracellular HBV clearance and induce apoptosis (16), but its overall response rate is low, and there are significant individual differences. However, the efficacy of IFN-α is relatively low, with only a subset of patients responding, and response rates vary among different CHB patients. The effectiveness of achieving an early response and sustained viral suppression through interferon therapy is influenced by multiple factors (17). Therefore, identifying key host factors regulating the anti-HBV effects of IFN-α is critically important. Increasing evidence highlights the therapeutic value of modulating intracellular signaling cascades alongside conventional antiviral therapies. Synergistic strategies combining interferons with pathway-specific agents may offer greater efficacy in HBV suppression (18).

It is well known that the liver, as a sexually dimorphic organ, possesses enzyme systems that convert estrogens into soluble metabolites for excretion. There are sex differences in the incidence and progression of various liver diseases (19). Sex is an important factor in clinical practice that affects HBV infection and disease severity (20, 21). Estrogen/estrogen receptors exhibit hepatoprotective effects, suppressing HBV transcription (22, 23) and its progression to hepatocellular carcinoma (HCC) (24–26). Studies have shown that women respond better to IFN-α during CHB treatment. When patients’ peripheral blood mononuclear cells (PBMCs) were cocultured with IFN-α, the levels of estrogen receptors increased with increasing concentrations of IFN-α (27). These findings indicate that estrogen/estrogen receptors may be involved in the inhibitory effects of IFN-α on HBV (28). In support of this, recent advances in 3D genome technologies have revealed that the spatial positioning of HBV covalently closed circular DNA (cccDNA) within the host nucleus may influence viral gene transcription and persistence, suggesting that nuclear architecture and chromatin context are integral to viral regulation (29). Therefore, the interaction mechanism by which the estrogen receptor ERα and IFN-α suppress HBV transcription and replication warrants further exploration.

Among guanylate-binding proteins, GBP1 was the first identified protein and can be induced for early expression by IFN-α. GBP1 has an N-terminal globular GTPase domain and a C-terminal CaaX motif, which enables partial or complete prenylation (30). Studies suggest that GBP1 is involved in antiviral and antitumor processes through inflammasomes, oxidative reactions, and autophagy (31, 32). Our previous research revealed that GBP1 may affect the efficacy of IFN-α against HBV, with elevated GBP1 expression associated with decreased HBsAg levels (33). However, whether GBP1 influences the interaction between ERα and IFN-α and enhances their inhibitory effects on HBV remains unclear.

This study aimed to investigate the role of GBP1 in the combined anti-HBV effects of ERα and pegylated IFNα-2b, as well as its interaction with ERα. Our results revealed that GBP1 serves as a cross-regulating gene for ERα and IFN-α, is highly expressed in the PBMCs and liver tissues of CHB patients, and is specifically positively correlated with ERα. Overexpression of ERα coupled with pegylated IFNα-2b stimulation increased GBP1 expression and inhibited HBV replication. Our data suggest that combination therapy with ERα and PEGylated IFNα-2b enhances the suppression of HBV replication, potentially through the upregulation of GBP1 expression and alterations in its cellular distribution. These findings may provide new research directions for the effective treatment of CHB patients.

## 2 Materials and methods

### 2.1 Participants

Sixty-six adult CHB patients (40 males and 26 females) treated with peg IFNα-2b were recruited at the infection department of the First Affiliated Hospital of Chongqing Medical University from October 2020 to December 2022. This study was authorized and approved by the Ethics Committee of the First Affiliated Hospital of Chongqing Medical University (application project number: 20221501). After obtaining informed consent from all subjects and their parents, they were included in the experiment. All the subjects signed the informed consent form. 34 patients who reached the clinical cure standard after treatment with peg IFNα-2b and 17 healthy controls were included simultaneously. The inclusion criteria included the following: (i) CHB confirmed for more than 6 months; (ii) HBeAg negative and HBsAg positive; (iii) HBV DNA levels below the lower limit of detection; (iv) patients in the treatment group without previous treatment for IFNα; (v) patients in the functional cure group who stopped for more than 3 months; (vi) HBsAb negative or positive; (vii) patients with no other disease history; and (viii) patients with CHB who met the criteria for interferon therapy published in ‘Chronic Hepatitis B Guidelines (2022)’. Samples of blood or liver tissue were taken from patients infected with HBV. Blood samples were also collected from healthy controls. qPCR or ELISA was used to confirm HBV infection in this study.

### 2.2 Analysis of serum HBV markers and liver function

Blood samples collected for the extraction of peripheral blood mononuclear cells (PBMCs) and serum separation were stored at 4°C. Serum alanine aminotransferase (ALT), aspartate aminotransferase (AST), white blood cell (WBC), total bilirubin (TB), and direct bilirubin (DB) levels were detected via an automatic analyzer (Roche Diagnostics, Switzerland). Biochemical immunoassays for the Architect-i2000 system were used to detect HBsAg levels in serum (Abbott Laboratories, USA). Serum GBP1 levels were measured via an ELISA kit (Jianglai, China). The levels of GBP1 and ERα in PBMCs and HBV DNA in serum were determined via qRT-PCR (Sansure Biotech, China).

### 2.3 Cell culture and virus infection

HepG2-NTCP cells were obtained from the infection laboratory of Chongqing Medical University, and the HepG2 2.15 and Huh7 cell lines were purchased from the Chinese Type Culture Collection (CTCC). HepG2-NTCP cells were cultured in Dulbecco’s modified Eagle’s medium (DMEM, Sigma, Germany) supplemented with 10% fetal bovine serum (FBS, Gibco BRL, USA), 100 U/ml penicillin, and 100 μg/ml streptomycin (Gibco BRL, USA) in an incubator with 5% CO_2_ at 37°C. HepG2 2.15 and Huh7 cell lines, treated with 400 μg/ml G418 (BioFroxx, Germany), were used to screen for the cell lines that produce hepatitis B virus to maintain viral replication. Following the manufacturer’s instructions, DNA transfection agents (Invitrogen, USA) were used to transfect the genes. In accordance with previous procedures, HepG2 NTCP cells were infected with 2 × 10^3/cell HBV-1.3 particles for 24 hours under 4% PEG 8000 and then cultured in standard medium supplemented with 2.5% DMSO after incubation (34). Transfection was performed after three washes with PBS, followed by drug intervention and extraction of cell RNA and protein.

### 2.4 Drugs and plasmids

IFNα-2b (Xiamen Tebao Company, China) was mixed, packed into a 1.5 ml EP tube and stored at 4°C. The 3×Flag-ERα, 3×Flag-GBP1, 3×GST-GBP1, 3×Flag-ERα-ΔAB, and 3xFlag-ERα-ΔEF plasmids were constructed by our laboratory. All the constructs were confirmed via DNA sequencing and western blotting. The overexpression vectors for ERα, pcDNA3.1, GBP1 short-chain interferon RNA (si-GBP1-1, si-GBP1-2), or nontargeted siRNA (si-control) were purchased from Beijing Entomobiology (Beijing Tsingke Biotech, China). After verification, the best titer was selected for subsequent experiments. ERα, pcDNA3.1, si-GBP1-1, si-GBP1-2, and si-control were transfected into HepG2-NTCP or Huh7 cells via Lipofectamine 3000 reagent (Invitrogen, USA) following the manufacturer’s instructions. The cells were harvested at the necessary times to detect viral markers. A list of primer sequences used in the experiment is provided in the appendix. HBsAg (NB100-62652, Novus, USA), HBcAg (ab8637, Abcam, Britain), anti-GST (66001-2, Prointech, China), anti-HBs (NB100-62652, Novus, USA), anti-β-actin (81115-1-RR, Prointech, China), anti-Flag (66008-4-Ig, Prointech, China), and anti-histone H3 (AF0009, Beyotime, China) were purchased.

### 2.5 Enzyme-linked immunosorbent assay

The cells were transfected with the DNA plasmid or siRNA or stimulated with peg IFNα-2b. HBsAg and HBeAg were detected in the supernatants of cultured cells via ELISA kits (KHB, China).

### 2.6 Western blot analysis

After plasmid transfection or drug treatment, the cells were collected and lysed with radioimmunoprecipitation assay (RIPA) buffer (Roche, Mannheim, Germany). A protein analysis reagent (Beyotime) was used to determine the protein concentration. For each sample, 30 μg of GOF protein was separated via SDS-polyacrylamide gel electrophoresis and transferred to a polyvinylidene fluoride membrane (GE Healthcare, Buckinghamshire, UK). A chemiluminescence reagent was used to develop the images. The density of the target protein band was normalized to that of β-actin. Each experiment was repeated three times.

### 2.7 Quantitative real-time PCR for RNA expression detection

TRIzol reagent (DP424, Tiangen, China) and a FastKing RT kit (KR116-02, Tiangen, China) were used to isolate total cellular RNA and synthesize cDNA. Total RNA was reverse transcribed via Fast SYBR™ Green Master Mix (1725122, Bio-Rad, USA) in a QuantStudio Six Flex (Thermo Fisher Scientific, USA). The expression level was standardized to that of β-actin, and three independent amplifications were performed for each sample. The relative expression of each target gene was calculated via the 2^-ΔΔCt^ approach.

### 2.8 Northern blot

Total RNA was electrophoresed on 2% denaturing agarose gels containing 1% formaldehyde, extracted from the indicated samples using TRIzol. SSC buffer was used to transfer samples to positively charged NC membranes. The membranes were UV-cross-linked at 265 nm (200,000 J/cm²) and prehybridized before being soaked in biotin-labelled probes at 65°C for 16–20 hours. In accordance with the manufacturer’s instructions, the Chemiluminescent Nucleic Acid Detection Module was used to detect biotin signals after the samples were washed with washing buffer. The probes used were junction sequences designed to detect only HBV RNA.

### 2.9 RNA-sequence

Huh7 cells were transfected with the overexpression vector ERα or pcDNA3.1 for 48 h or stimulated with 1,000 U/ml peg IFNα-2b or absolute alcohol for 24 h, respectively. The cells were collected for RNA-seq analysis to screen the intersecting genes among the DEGs in the ERα-overexpressing group and the PEG IFNα-2b-stimulated group. Differentially expressed genes were analyzed and identified via the BGI online platform (https://biosys.bgi.com) (35). The intersecting genes were selected to generate Venn diagrams, volcano plots, and heatmaps.

### 2.10 Coimmunoprecipitation assay

The 3xFlag-ERα and 3xGST-GBP1 plasmids were cotransfected into HepG2 2.15 cells for 48 h. Cell samples were lysed with RIPA lysis buffer, and the resulting cell lysates were separated. Lysates were immunoprecipitated with A+G magnetic beads (88802, Thermo Fisher Scientific) labeled with an anti-GBP1 antibody (15303-1-AP, Prointech, China) or rabbit/mouse immunoglobin G, where the IgG antibody was used as a negative control. A small amount of precleared lysate (input) was saved for later analyses, and the remainder was divided equally among the GBP1- and IgG-coated beads for immunoprecipitation (IP). Immunoblotting (IB) was performed using the indicated antibodies after the beads were washed and eluted with reducing sample buffer (Thermo Fisher Scientific).

### 2.11 Molecular docking

The receptor proteins corresponding to ERα and GBP1 were downloaded from the PDB database and processed. AutoDock Tools 1.5.6 and PyMOLW software were used for molecular docking and visualization (36).

### 2.12 Molecular dynamics simulations

Based on the molecular docking results, molecular dynamics simulations were performed using Gromacs 2022.3 to investigate the key interaction between ERα and GBP1 (37–39). The LEAP module was used to obtain the initial topology file. The GAFF force field was employed for amino acid detection. The water model used was TIP3P, and Na+ ions were added to ensure the system’s electrical neutrality. The cutoff value of the box was set to 10 Å. The system was minimized using the steepest descent method and the conjugate gradient method, and then equilibrated via the canonical ensemble (NVT) and the isothermal-isobaric ensemble (NPT). The molecular dynamics simulations were carried out for 100 ns at ambient temperature and pressure. Tracks of the root mean square deviation (RMSD), root mean square fluctuation (RMSF), radius of gyration (Rg), solvent accessible surface area (SASA), and number of hydrogen bonds.

### 2.13 Immunofluorescence assay

After transfection, the cells were grown on glass slides for 48 h and then fixed in 4% polyethylene glycol (PEG) methanol. Anti-GBP1 (15303-1-AP, Prointech, China), anti-ERα (8644, CST, USA), and anti-FLAG-Tag (monoclonal mouse Flag, 66008-3, murine IgX, Proteintech) were diluted at a 1:1 ratio and incubated overnight. Afterwards, the cells were rinsed with PBS and treated with a fluorescein-coupled secondary antibody (Alexa Fluor 488 to 555 donkey-a-m/Rbg antibody; Beyotime) for 2 hours. After being stained with DAPI, the cells were dried. Images were obtained via NIS-Viewer software (Nikon A1, Japan).

### 2.14 Immunohistochemical staining

Liver tissue samples were fixed overnight in a 4% paraformaldehyde solution and then dehydrated using a gradient of alcohol concentrations. After 1 hour of xylene transparency, paraffin-embedded sections were made. Then, the samples were dewaxed and hydrated for antigen repair and incubated with H_2_O_2_ solution at room temperature for 10 minutes to block endogenous peroxidase activity. Following blocking, the sections were incubated overnight at 4°C with primary antibodies. After the samples were washed with PBS, they were incubated with HRP-conjugated secondary antibodies for 1 h. DAB staining was then performed, followed by hematoxylin restaining, dehydration, and xylene transparency. Images were obtained via a microscope (3DHistech, Budapest, Hungary).

### 2.15 Bioinformatics

*The* RNA sequences from the liver tissues of CHB patients were entered into the Metascape database platform, with the species set to human. Bubble maps of enriched signalling pathways were generated via Kyoto Encyclopedia of Genes and Genomes (KEGG) enrichment analysis.

### 2.16 Statistical analysis

For statistical analysis, R, RStudio, GraphPad Prism 9.3.1, and MedCalc software were used. The data are presented as the means ± standard. Multigroup comparisons were conducted via one-way analysis of variance, whereas two-sample comparisons were conducted via Student’s t tests. An analysis of correlation factors was performed via Spearman correlation analysis. Receiver operating characteristic (ROC) curves were used to evaluate the value of GBP1 and ERα expression in assessing the early response to peg IFNα-2b therapy. *P* < 0.05 was considered to indicate a significant difference.

## 3 Results

### 3.1 ERα and peg IFNα-2b inhibit HBV transcription and replication

**Figure 1:**
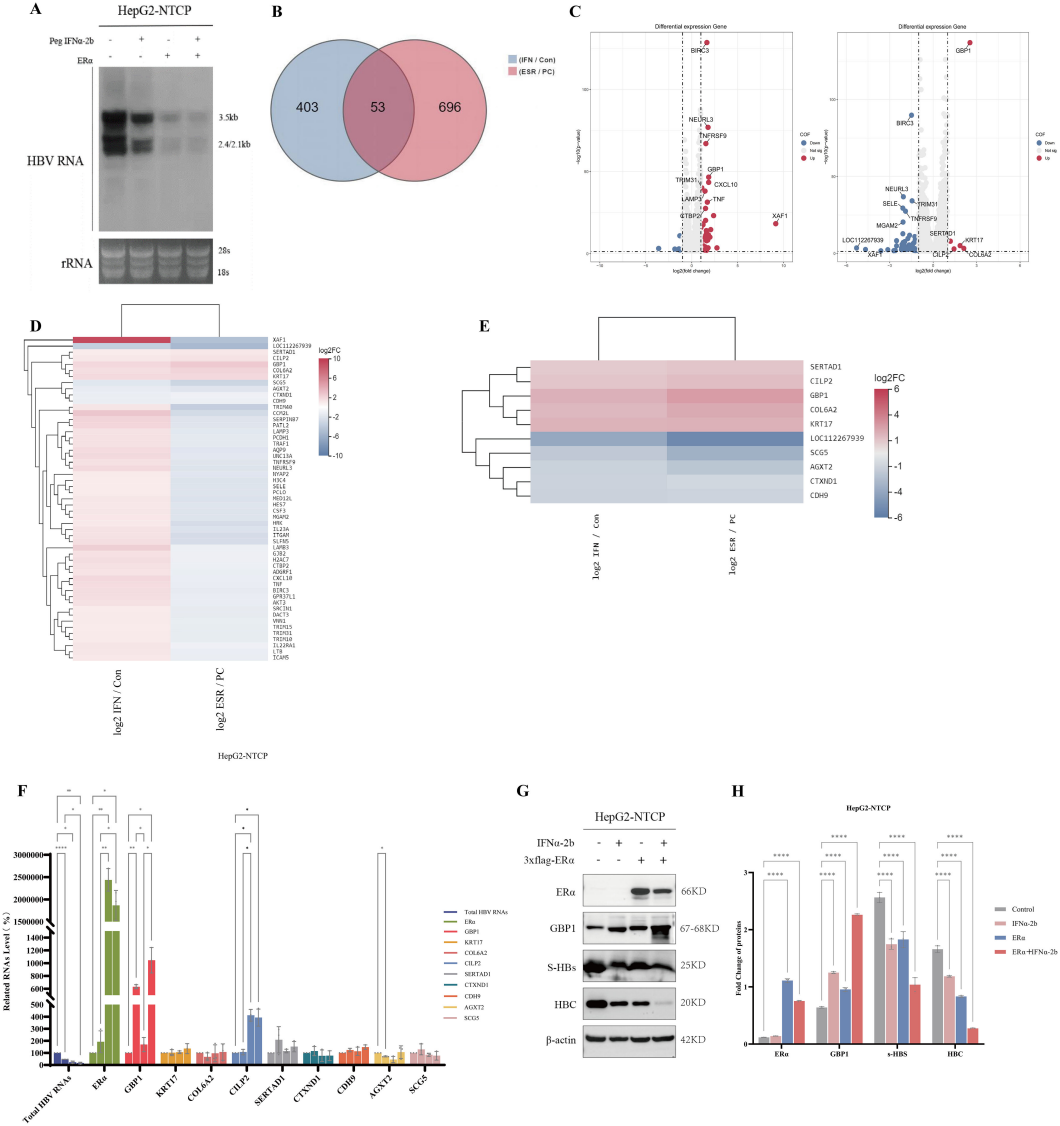
ERα and peg IFNα-2b inhibited HBV transcription and replication, and ERα or peg IFNα-2b induced GBP1 gene expression. **(A)** The overexpression vector ERα and 1,000 U/ml peg IFNα-2b were used to treat HepG2-NTCP cells infected with HBV for 48 h, and then the levels of HBV RNA expression were observed via Northern blotting. **(B)** Venn diagrams of differentially expressed genes identified through the intersection of the ERα-overexpressing group and the PEG IFNα-2b-stimulated group. **(C)** Volcano plots showing the adjusted p-value for intersectional genes between the ERα-overexpressing group and the IFNα-2b-stimulated group (log2FC ≥ 1, adjusted P < 0.05). **(D)** Heatmap of the intersection genes; blue indicates lower expression, and red indicates higher expression. **(E)** Heatmap of nine genes whose expression was simultaneously up- or downregulated; blue indicates lower expression, and red indicates higher expression. **(F)** The expression of nine genes was determined by qRT-PCR in HBV-infected HepG2-NTCP cells. **(G)** The levels of ERα, GBP1, the HBV surface protein, and the HBV core protein were examined in HBV-infected HepG2-NTCP cells that were treated with ERα or peg IFNα-2b alone or in combination. All the data are presented as the means ± SDs; *P < 0.05, **P < 0.01, ****P < 0.0001.

The inhibitory effect of peg IFNα-2b on HBV transcription has been widely recognized and studied. Previous studies have indicated that Erα inhibits HBV transcription. To analyze the effect of combining ERα and peg IFNα-2b on the HBV life cycle, we used the overexpression vector ERα and 1,000 U/ml peg IFNα-2b to intervene in HBV-infected HepG2-NTCP cells and observed changes in HBV RNA expression. As shown by our results, treatment with the overexpression vector ERα or peg IFNα-2b alone or in combination resulted in a notable decrease in HBV RNA expression. Interestingly, overexpressing ERα via cotreatment with peg IFNα-2b further reduced HBV RNA levels (**Figure 1A**). These results indicate that ERα combined with peg IFNα-2b may inhibit HBV transcription and replication.

### 3.2 Screening and validation of intersecting genes with ERα and peg IFNα-2b

We then used RNA-seq to identify the intersectional genes of differentially expressed genes in the ERα overexpression group and the IFNα-2b-stimulated group, and analyzed them using the BGI online platform. As shown in **Figure 1B**, there were 456 DEGs in the ERα-overexpressing group, 749 DEGs in the IFNα-2b-stimulated group, and 53 intersecting genes in the two groups (log_2_FC≥1, adjusted *P* < 0.05). The expression of 53 intersectional genes in the ERα-overexpressing group did not completely correspond with that in the PEG IFNα-2b-stimulated group (**Figures 1C, D**). To further analyze the synergism between the two groups, we screened nine genes whose expression was simultaneously up- or downregulated, namely, SERTAD1, CILP2, GBP1, COL6A2, KRT17, SCG5, AGXT2, CTXND1, and CDH9 (**Figure 1E**). We also overexpressed ERα or peg IFNα-2b alone or in combination in HepG2-NTCP cells infected with HBV to verify the sequencing results, and the expression of the nine genes described above and total HBV RNA were analyzed via qRT-PCR. After stimulation with ERα or peg IFNα-2b alone or in combination, the gene expression of GBP1, CILP2, and AGXT2 significantly changed, and only GBP1 was the most significantly upregulated gene. In addition, the level of total HBV RNA was significantly reduced (**Figure 1F**). Next, we evaluated the influence of ERα and peg IFNα-2b on HBV replication. HepG2-NTCP cells infected with HBV overexpressing ERα or peg IFNα-2b alone or in combination were cultured. The results revealed that ERα and GBP1 expression were considerably elevated, while S-HBs and HBc decreased significantly. Furthermore, the combination of ERα with peg IFNα-2b had a more significant treatment effect (**Figure 1G**). The above results suggest that ERα and peg IFNα-2b exert their antiviral effects by increasing GBP1 expression.

### 3.3 ERα interacts with GBP1

**Figure 2:**
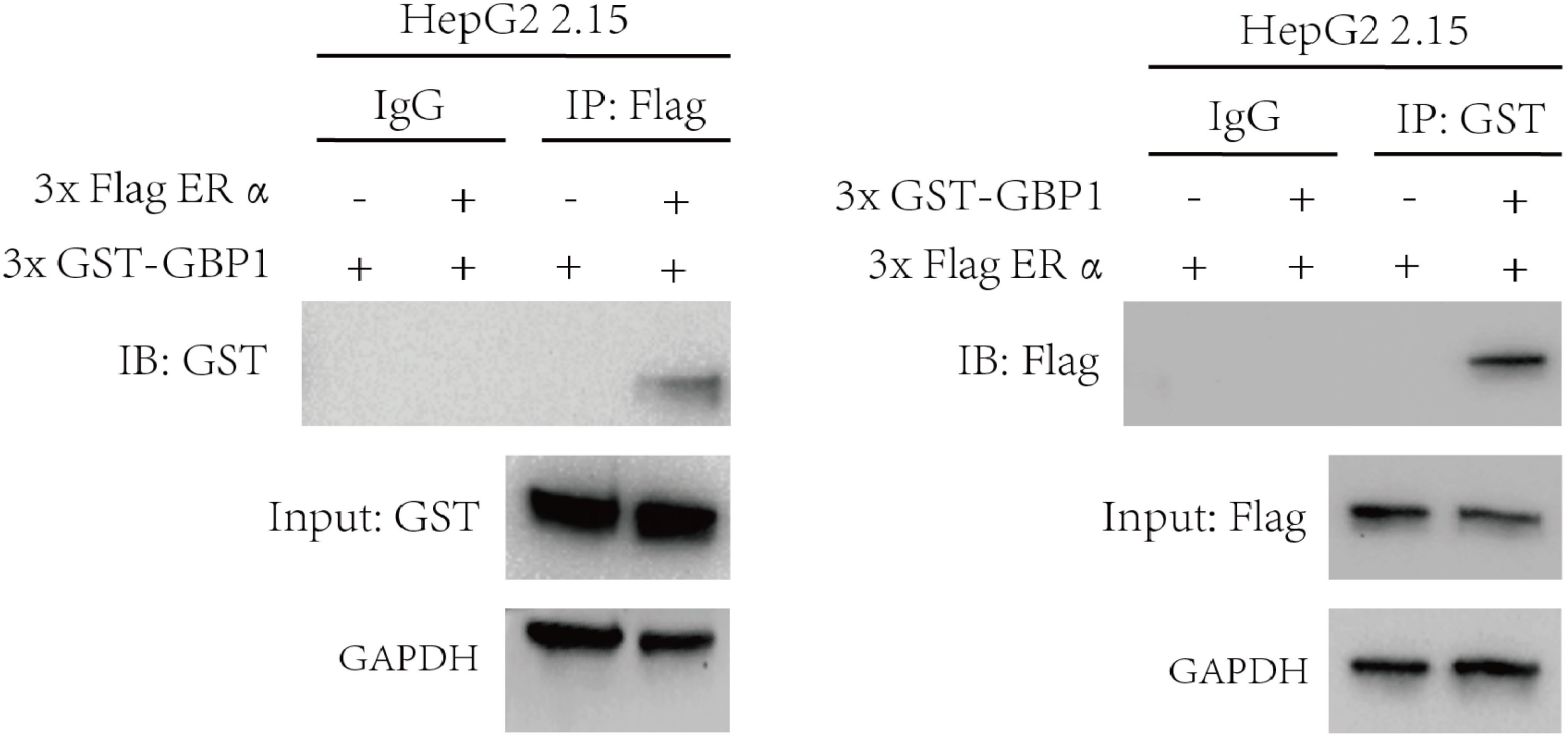
Physical interaction between endogenous GBP1 and ERα. The 3xFlag-ERα and 3xGST-GBP1 plasmids were cotransfected into HepG2 2.15 cells for 48 h. Using anti-GST/anti-Flag antibodies, a co-IP assay was performed on lysates and precipitated samples.

Based on our previous studies, we found that pegylated IFNα-2b-induced GBP1 suppresses HBV replication. However, the interaction of GBP1 with ERα has rarely been reported. The interaction between ERα and GBP1 was demonstrated via a coimmunoprecipitation (Co-IP) assay in HepG2 2.15 cells (**Figure 2**).

### 3.4 ERα and peg IFNα-2b induce GBP1 expression and enhance its anti-HBV activity

**Figure 3:**
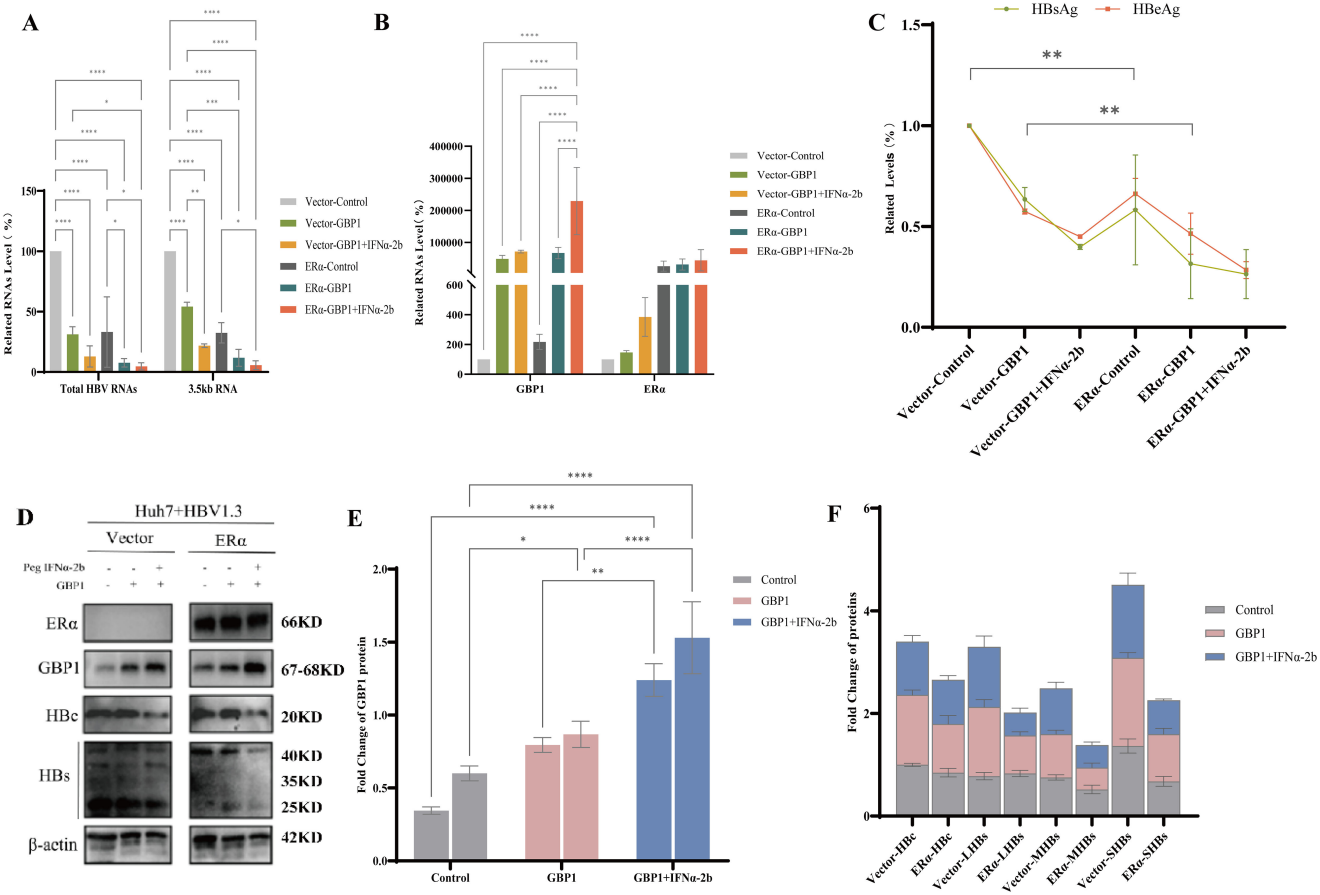
ERα and peg IFNα-2b promoted GBP1 expression and enhanced its anti-HBV activity in Huh7-HBV1.3 cells. Vector- and ERα-transfected Huh7-HBV1.3 cells were stimulated with a GBP1 overexpression plasmid and 1,000 U/ml peg IFNα-2b. **(A, B)** The mRNA levels of total HBV RNA, 3.5 kb RNA **(A)**, ERα, and GBP1 **(B)** were analyzed via qRT-PCR. **(C)** ELISA was used to determine the levels of HBsAg and HBeAg. **(D-F)** The levels of GBP1 **(E)**, the HBV core protein, and the HBV surface protein **(F)** were examined via Western blotting. All the data are presented as the means ± SDs; *P < 0.05, **P < 0.01, ***P < 0.001, ****P < 0.0001.

To further verify the inhibitory effect of GBP1 induced by ERα and peg IFNα-2b on HBV replication, we constructed Huh7-HBV1.3 cells with stable ERα expression. Vector- and ERα-transfected Huh7-HBV1.3 cells were stimulated with a GBP1 overexpression plasmid and 1,000 U/ml peg IFNα-2b. After GBP1 overexpression or combination with peg IFNα-2b, the transcription levels of total HBV RNA and 3.5 kb RNA were significantly reduced. The reduction was even greater in the ERα group (**Figure 3A**). GBP1 was notably increased and increased more in the ERα group than in the control group. At the same time, the expression of ERα was also increased (**Figure 3B**). We then verified the changes in the expression of GBP1, HBs, and HBc at the protein level in the vector and ERα groups. The results were largely consistent with those obtained by PCR (**Figures 3D-F**). In addition, HBsAg and HBeAg secretion were significantly inhibited after GBP1 overexpression or combination with peg IFNα-2b (**Figure 3C**). Taken together, these results indicate that ERα and peg IFNα-2b induce GBP1 expression and enhance its anti-HBV activity.

### 3.5 GBP1 can modulate the anti-HBV activity of peg IFNα-2b and Erα

**Figure 4:**
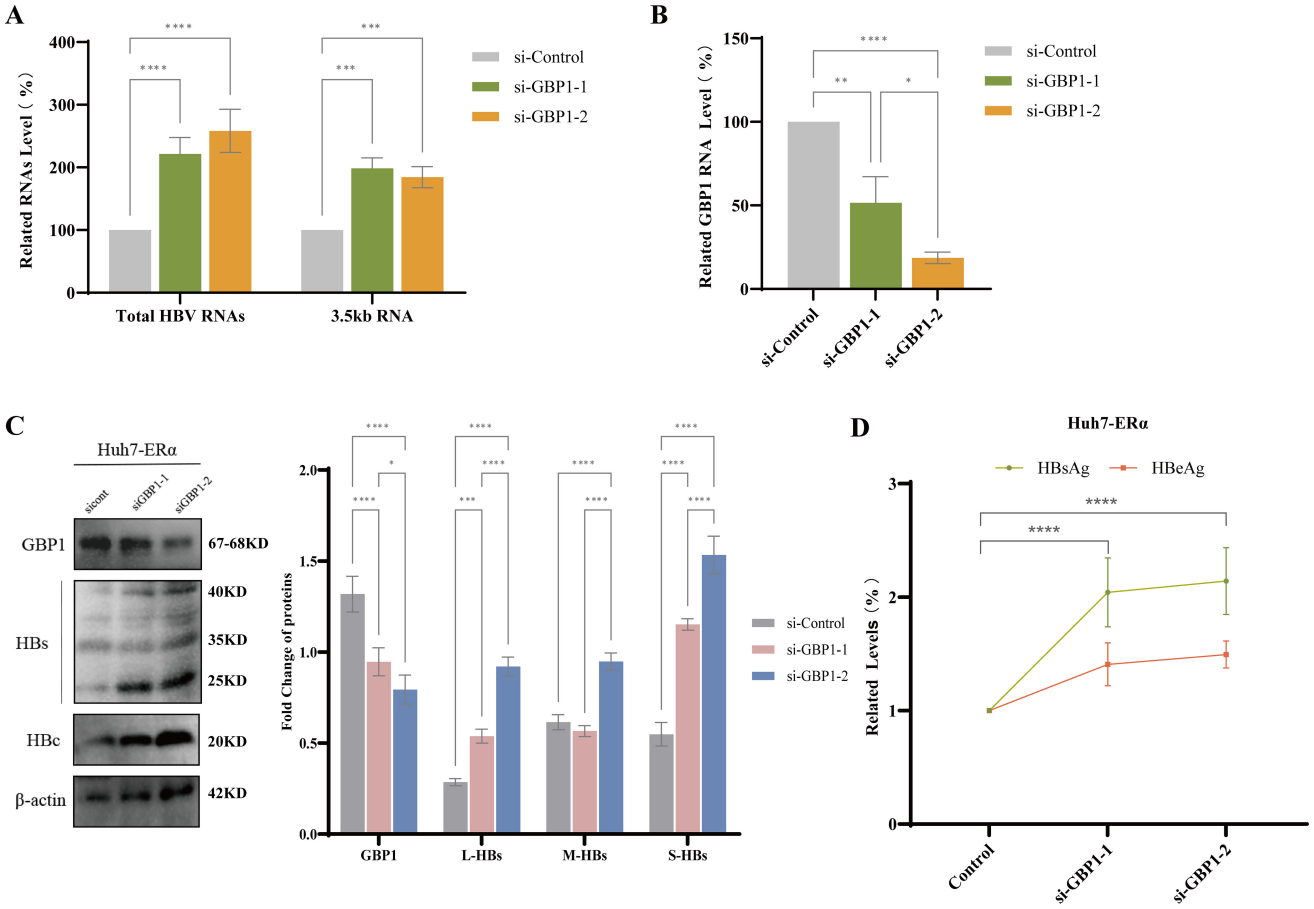
GBP1 silencing promoted HBV transcription and replication. Huh7-ERα cells were transduced with specific siRNAs (si-control, si-GBP1-1, and si-GBP1-2) to silence endogenous GBP1. **(A, B)** qRT-PCR was utilized to assess the mRNA levels of total HBV RNA, 3.5 kb RNA **(A)**, and GBP1 **(B, C)** Western blotting was used to determine the levels of GBP1, the HBV surface protein, and the HBV core protein. **(D)** ELISA was used to determine the levels of HBsAg and HBeAg. All the data are presented as the means ± SDs; *P < 0.05, **P < 0.01, ***P < 0.001, ****P < 0.0001.

**Figure 5:**
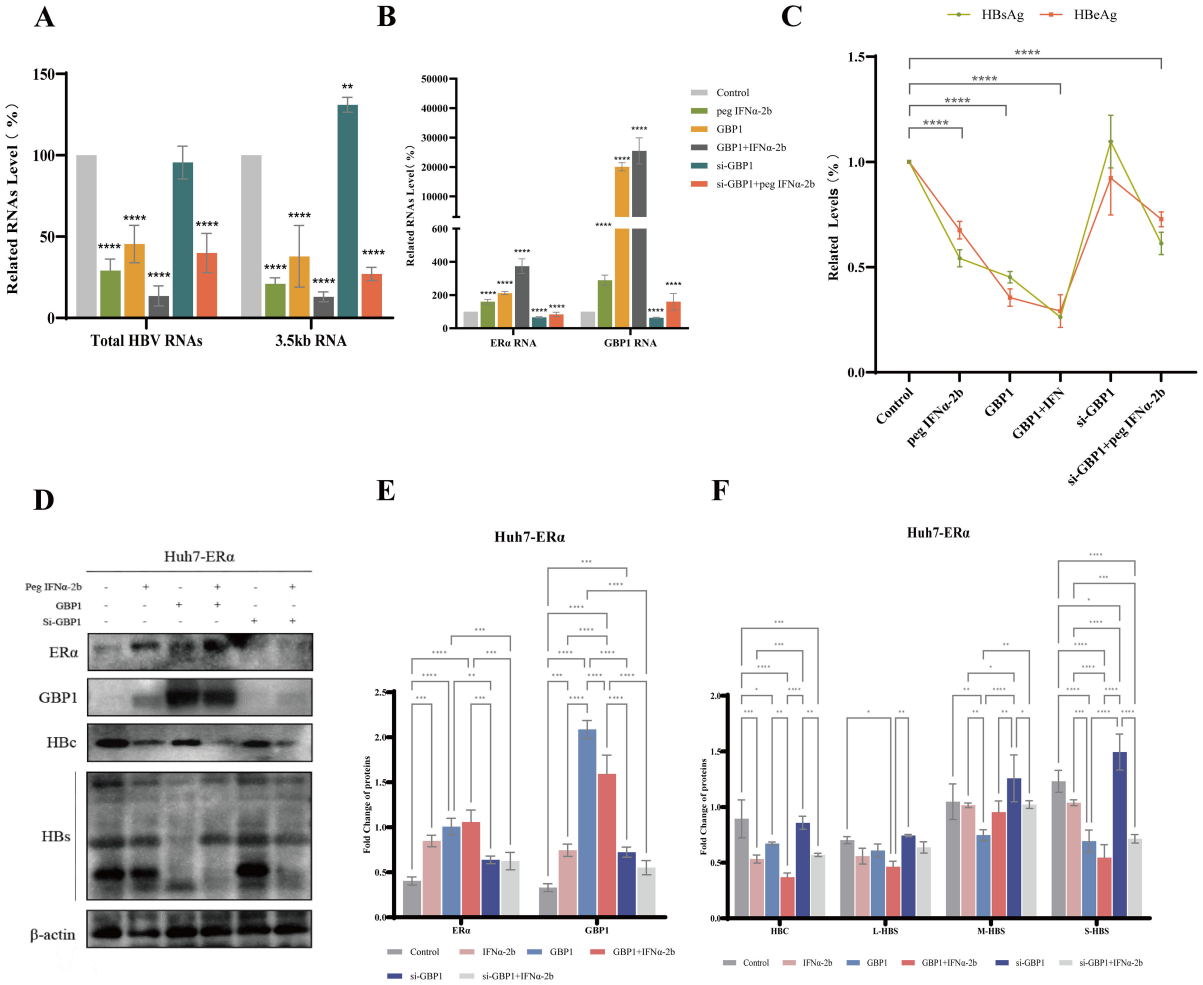
GBP1 potentiated the antiviral effects of peg IFNα-2b and ERα. Huh7-ERα cells were transfected with the wild-type hGBP1 plasmid or si-GBP1 and subsequently cotreated with 1,000 U/ml peg IFNα-2b. **(A, B)** qRT-PCR was performed to analyze the mRNA levels of total HBV RNA, 3.5 kb RNA **(A)**, ERα, and GBP1 **(B, C)** ELISA was used to determine the levels of HBsAg and HBeAg. **(D)** The levels of ERα, GBP1, the HBV surface protein, and the HBV core protein were examined via Western blotting. All the data are presented as the means ± SDs; *P < 0.05, **P < 0.01, ***P < 0.001, ****P < 0.0001. **(E, F)** The levels of ERα, GBP1, HBV surface protein and HBV core protein were detected by Western blotting.

We have shown that ERα and peg IFNα-2b induce GBP1 expression and enhance its anti-HBV activity. To further analyze the regulatory effects of GBP1 on ERα and peg IFNα-2b, we constructed Huh7-HBV1.3 cells with stable ERα expression. GBP1 short-chain interferon RNA (si-GBP1-1, si-GBP1-2) or nontargeted siRNA (si-control) was used to silence GBP1 expression in Huh7-ERα cells specifically. *In vitro*, the silencing of GBP1 increased HBsAg and HBeAg secretion (**Figure 4D**). Transfection with siRNA constructs targeting GBP1 (si-GBP1) reduced GBP1 mRNA and protein expression relative to that in the si-control group but upregulated total HBV RNA, 3.5 kb RNA, HBc protein, and HBs protein expression (**Figures 4A-C**). To elucidate the function of GBP1 in peg IFN-2b and the ER against infection, Huh7-ERα cells were transfected with the wild-type hGBP1 plasmid or si-GBP1 and subsequently cotreated with 1,000 U/ml peg IFNα-2b. Our results revealed that overexpression of GBP1 inhibited HBsAg and HBeAg production; decreased the levels of total HBV RNA, 3.5 kb RNA, HBc protein, and HBs protein; and increased ERα and GBP1 expression in Huh7-ERα cells, whereas silencing of GBP1 inhibited GBP1 against viral infection and reduced ERα and GBP1 expression (**Figures 5A-D**). Notably, GBP1 overexpression, in combination with peptidation using IFNα-2b, further confirmed the previous conclusion. Moreover, our results showed that silencing GBP1, in combination with peg IFNα-2b, partly restored the inhibitory effect of IFNα-2b on HBV (**Figures 5A-D**). These findings further suggest that GBP1 seems to have a beneficial effect on the control of ERα and peg IFNα-2b against HBV infection.

### 3.6 ERα could function by binding to GBP1

**Figure 6:**
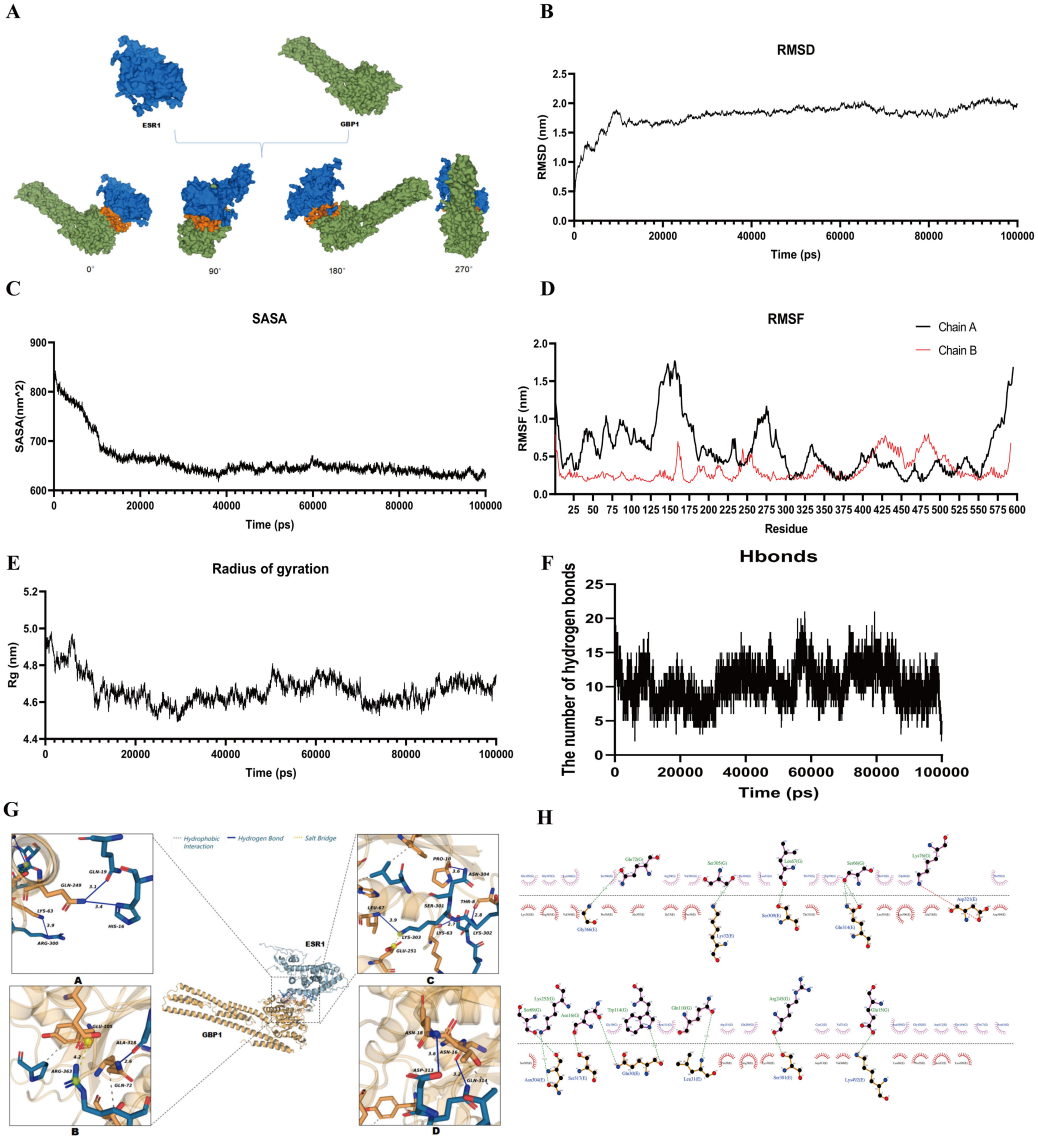
ERα functions by binding to GBP1. Molecular docking and MD simulations were performed to determine the complex stability and action sites of ERα and GBP1. **(A)** Modules of the Erα and GBP1 complex. **(B-F)** Results of the molecular dynamics simulations. Tracks of the RMSD **(B)**, SASA **(C)**, RMSF **(D)**, radius of gyration **(E)**, and number of hydrogen bonds **(F, G)** Molecular docking of ERα with the GBP1 protein. **(H)** Interacting amino acid residues of ERα and GBP1.

The above results implied that ERα and GBP1 interact. To further verify our hypothesis, we employed molecular dynamics simulations and molecular docking to investigate the stability and action sites of ERα and GBP1. First, hGBP1 (PDB: P32455) from the wild-type (WT) and ERα (PDB: P03372) from the wild-type (WT) were obtained from the protein database UniProt. HDOCK protein docking software was used to generate the protein structure with the highest combined score and confidence, as well as the lowest binding energy. The lower the energy was, the more stable the docking result was (**Figure 6A**). The effects were then simulated via 100 ns molecular dynamics simulations. As shown in **Figure 6B**, after approximately 20,000 ps of simulation, the system had essentially reached equilibrium, and the RMSD values stabilized at around 2.0 Å. The SASA values of the complex gradually stabilized after fluctuating for the first 40,000 ps and finally stabilized at approximately 650 mm^2^ (**Figure 6C**). The structure is mapped with RMSF values to indicate regional flexibility.

A larger RMSF value indicates a more flexible region and worse stability of the amino acids being analyzed. After 300, the RMSF values of the amino acid residues decreased, and the combination of the two was more stable (**Figure 6D**). Rg is an index used to evaluate the tightness of the overall structure of protein molecules. As shown in **Figure 6E**, the results revealed that the Rg values tended to be stable after 20,000 ps, indicating that the overall structure was compact, suggesting a tight overall design of the complex. Hydrogen bonds and hydrophobic interactions play essential roles in preserving protein conformation. The number of hydrogen bonds in the complex was determined from the Hbonds diagram (**Figure 6F**). Hydrogen bonds are considered to be the most important of all directed covalent interactions and can improve protein binding affinity. We identified the key amino acid sites involved in the interaction between ERα and GBP1 through molecular docking (**Table 1**). Our results revealed a higher number of hydrogen bonds between GBP1 and ERα, suggesting a greater stability of their interaction. The action sites of GBP1 and ERα were mainly concentrated in the EF region of ERα; some of the action sites were in the AB region, and the RMSF values suggested that they were in the EF region (**Figures 6G, H**). According to its structure and function, ERα is roughly divided into four regions: AB, C, D, and EF, and the AB region contains ligand-independent activation function 1 (AF-1). This functional region is not dependent on the activation of ligands (i.e., estrogen) and may be involved in regulating the transcription of estrogen-responsive genes by regulating estrogen receptor binding. The EF region is referred to as the ligand binding domain (LBD). The EF region contains a ligand-dependent activation function 2 (AF-2) that plays a critical role, including sensitivity to estrogen, receptor dimerization, nuclear localization, and influencing the activities of coactivators or coinhibitors (40). These findings demonstrate that the EF region of ERα is a crucial binding site for GBP1.

**Table 1:** Interacting amino acid residues of Erα and GBP1.

Hydrophobic interactions between ERα-GBP1 protein-protein complexes				Hydrogen bonding between ERα-GBP1 protein-protein complexes				Salt bridges between ERα-GBP1 protein-protein complexes			
Index	ERα residue	Distance	GBP1 residue	Index	ERα residue	Distance	GBP1 residue	Index	ERα residue	Distance	GBP1 residue
1	ASN-27	3.98	TYR-144	1	LEU-26	3.15	ASN-109	1	LYS-492	3.19	GLU-15
2	LYS-32	3.69	ILE-304	2	ARG-28	3.03	ASN-109				
3	LEU-306	3.37	HIS-74	3	GLN-30	2.75	ASN-16				
4	LEU-306	3.48	GLN-72	4	GLN-30	2.38	ASN-111				
5	THR-311	3.07	TRP-79	5	LEU-31	2.25	GLN-110				
6	ASP-313	3.91	TRP-79	6	LYS-32	3.15	MET-22				
7	GLN-314	2.73	TRP-79	7	SER-301	2.4	ARG-245				
8	LEU-320	0.65	THR-17	8	LYS-302	2.42	GLN-249				
9	ARG-363	3.42	GLU-105	9	ASN-304	2.03	LYS-252				
10	VAL-368	3.26	VAL-71	10	ASN-304	2.43	LYS-252				
				11	LEU-306	3.42	THR-70				
				12	ALA-307	3.5	THR-70				
				13	SER-309	2.46	LEU-67				
				14	THR-311	3.57	HIS-74				
				15	ASP-313	2.75	GLU-15				
				16	GLN-314	3.13	TRP-79				
				17	GLN-314	1.85	SER-66				
				18	SER-317	3.28	ASN-16				
				19	SER-317	3.03	GLY-77				
				20	LEU-320	3.25	ASN-18				
				21	ASP-321	2.75	LYS-76				
				22	ARG-363	2.31	LYS-106				
				23	VAL-364	1.67	GLN-72				
				24	GLY-366	1.82	GLN-72				

### 3.7 The EF region of Erα inhibits HBV replication through targeting GBP1.

**Figure 7:**
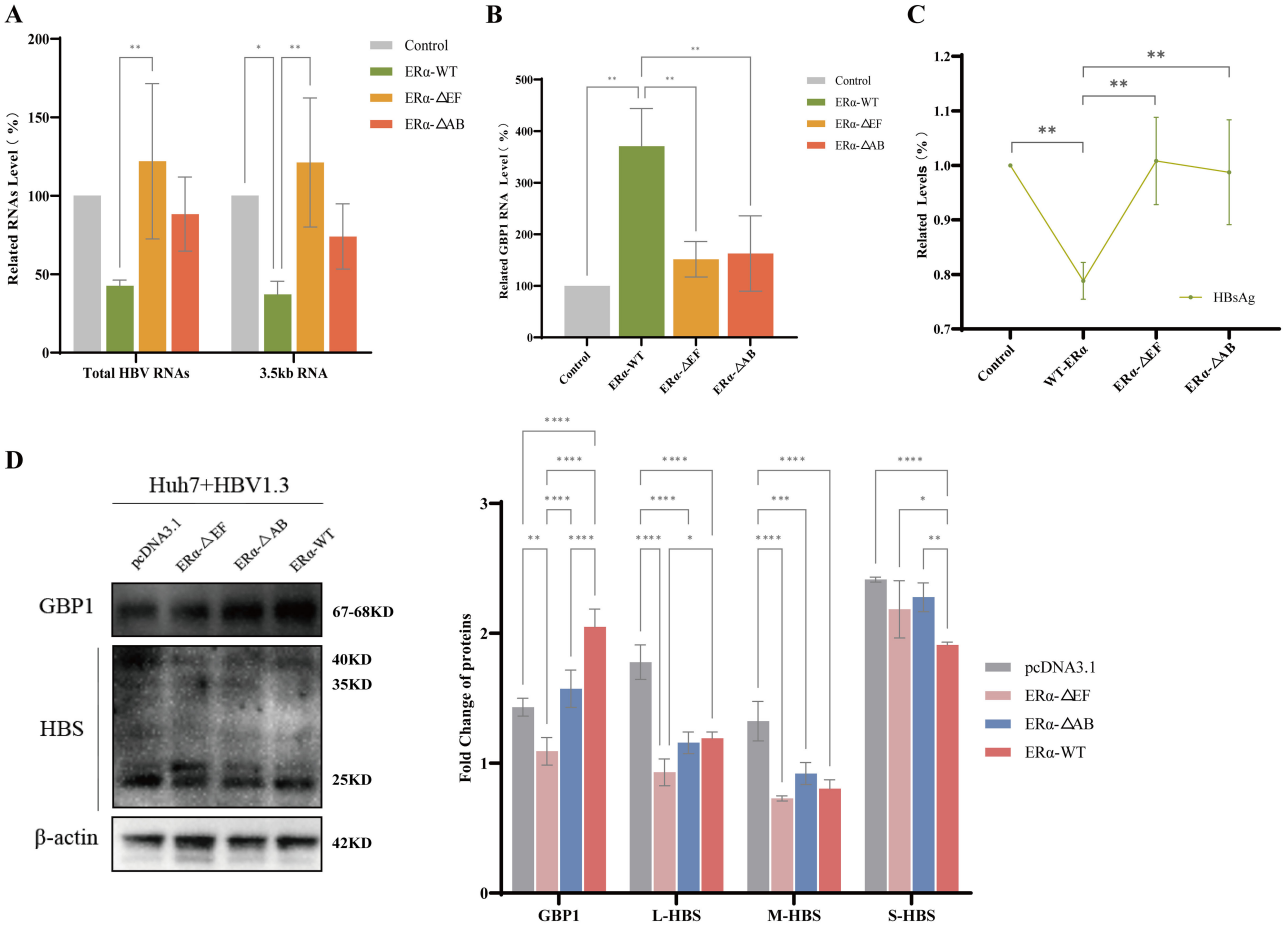
The EF region of ERα inhibited HBV replication through targeting GBP1. Huh7-HBV1.3 cells were transfected with pcDNA3.1, 3xFlag ERα-WT, 3xFlag ERα-ΔAB, and 3xFlag ERα-ΔEF plasmids, and then virological indicators were measured. **(A, B)** qRT-PCR was utilized to assess the mRNA levels of total HBV RNA, 3.5 kb RNA **(A)**, and GBP1 **(B, C)** HBsAg content was determined via ELISA. **(D)** The levels of GBP1 and HBV surface proteins were examined via Western blotting. All the data are presented as the means ± SDs; *P < 0.05, **P < 0.01, ***P < 0.001, ****P < 0.0001.

Combined with the above results, it was speculated that the AB or EF regions are the key regions through which ERα interacts with GBP1. Next, we investigated whether the AB and EF regions of ERα inhibited HBV replication by targeting GBP1 via direct binding. We transfected Huh7-HBV1.3 cells with pcDNA3.1, 3xFlag ERα-WT, 3xFlag ERα-ΔAB, and 3xFlag ERα-ΔEF plasmids and then measured virological indicators. Our results showed that, compared with ERα-WT, ERα-ΔAB and ERα-ΔEF offset the ERα-mediated inhibition of HBV replication, resulting in increased total HBV RNA and 3.5 kb RNA levels. Notably, ERα-ΔEF had a more significant effect. Compared with the control group, the ERα-△EF group presented even higher levels of total HBV RNA and 3.5 kb RNA. Moreover, overexpression of ERα promoted the expression of GBP1, and truncation of the AB and EF regions of ERα decreased GBP1 expression, with the EF region being the most significant (**Figures 7A, B**). Similar results were further observed at the HBsAg level (**Figure 7C**). Western blot analysis verified the effect of truncation of the AB and EF regions of Erα on GBP1 and HBs at the protein level, and the results were also consistent with the above findings (**Figure 7D**). These data suggest that the EF region is the primary region in which ERα engages in binding with GBP1.

### 3.8 Overexpression of ERα increases the expression of GBP1 in the nucleus

**Figure 8:**
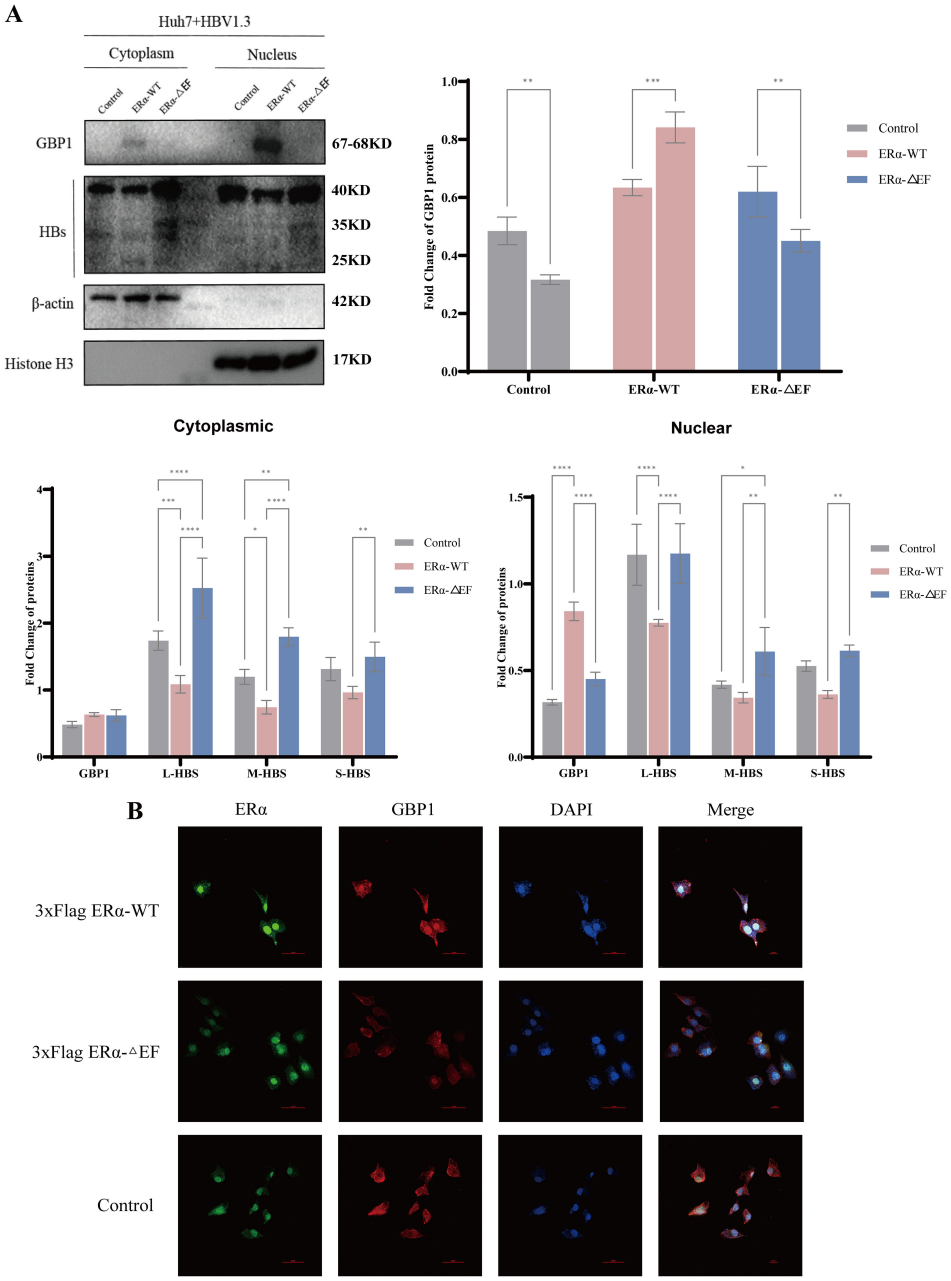
The overexpression of ERα increased the expression of GBP1 in the nucleus. Huh7-HBV1.3 cells were transfected with the pcDNA3.1, 3xFlag ERα-WT, or 3xFlag ERα-ΔEF plasmid, after which virological indicators were measured. **(A)** Western blot analysis revealed GBP1 and HBV surface protein levels in the nuclear and cytoplasmic fractions. **(B)** Immunofluorescence labeling with monoclonal anti-ERα (green) and anti-GBP1 (red) antibodies revealed the endogenous localization of the proteins in Huh7-HBV1.3 cells. Nuclei were counterstained with DAPI (blue). Scale bar: 50 μm. All the data are presented as the means ± SDs; *P < 0.05, **P < 0.01, ***P < 0.001, ****P < 0.0001.

ERα is known to act as a nuclear receptor. ERα is expressed mainly in the nucleus, whereas GBP1 is localized mainly in the cytoplasm. Based on the interaction between ERα and GBP1, we aimed to investigate whether this binding affects their subcellular localization. To this end, we extracted cytoplasmic and nuclear proteins from 3xFlag ERα-WT and 3xFlag ERα-ΔEF plasmid-transfected Huh7-HBV1.3 cells and control cells, respectively, and then examined the changes in the expression of GBP1 and HBs. After overexpressing ERα, we observed the protein abundance of GBP1 in the cell nucleus increases, surpassing that of GBP1 in the cytoplasm (**Figure 8A**). We subsequently determined the intracellular distributions of ERα and GBP1 via immunofluorescence. We found that ERα was located mainly in the nucleus. GBP1 was more highly expressed in the cytoplasm of the control group than in the nucleus. These results are in agreement with those of previous studies. The upregulation of ERα resulted in an increase in GBP1 expression within the nucleus, whereas the upregulation of ERα-ΔEF did not exert a substantial effect on GBP1 expression within the nucleus (**Figure 8B**). Therefore, we speculate that ERα increases the protein abundance of GBP1 in the nucleus and may even induce the nuclear translocation of GBP1.

### 3.9 ERα and GBP1 predict the response to peg IFNα-2b treatment in patients with CHB

**Figure 9:**
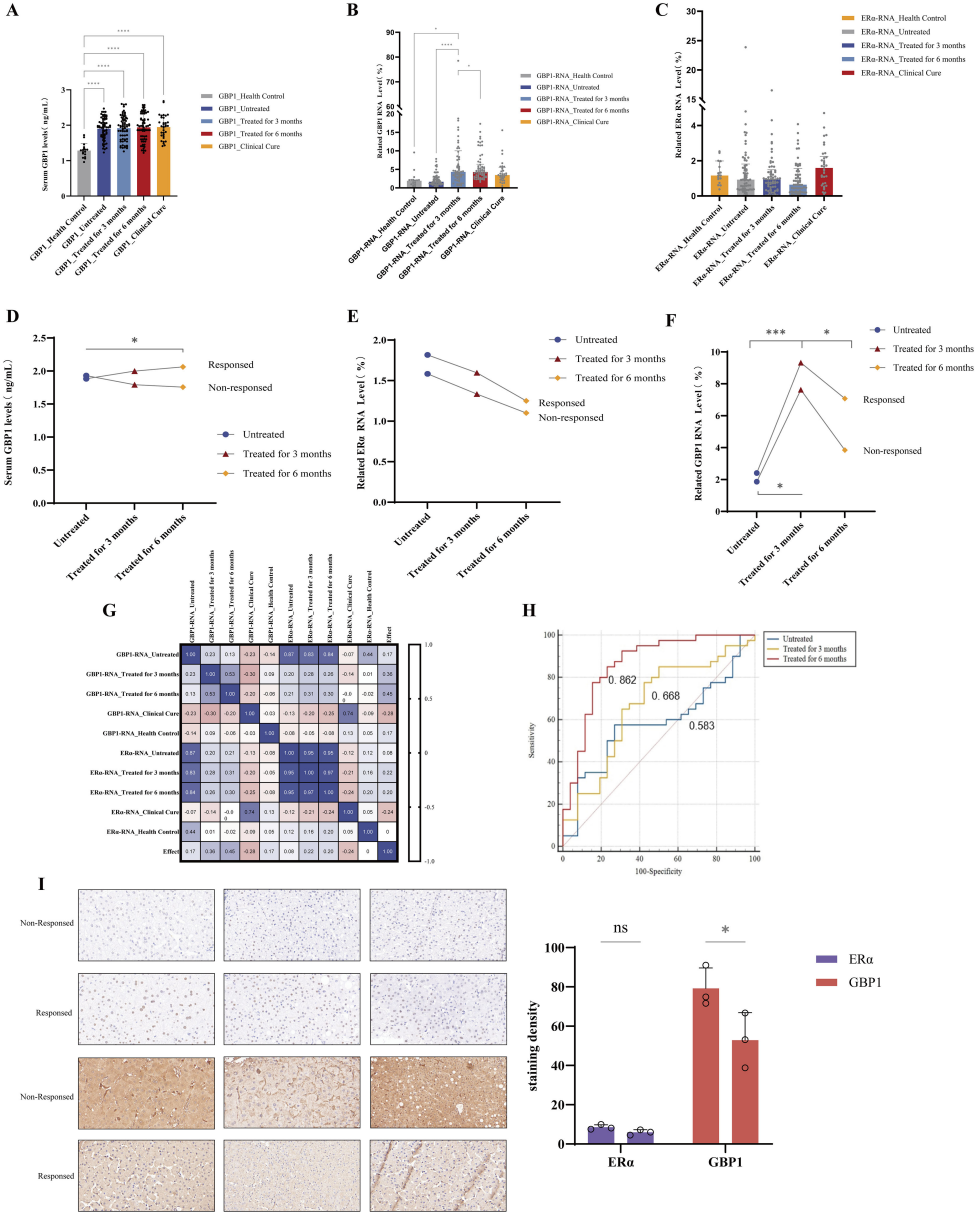
Changes in GBP1 and ERα in the peripheral blood of CHB patients treated with peg IFNα-2b. **(A-C)** Relative expression levels of GBP1 and ERα in CHB patients treated with peg IFNα-2b and healthy controls. Serum levels of GBP1 **(A)** were detected by ELISA. qRT-PCR was performed to detect GBP1 **(B)** and ERα **(C)** RNA expression **(A, D-F)** Relative expression levels of GBP1 and ERα in nonresponsive and responsive patients. Serum levels of GBP1 **(D)** were detected by ELISA. qRT-PCR was performed to detect ERα **(E)** and GBP1 **(F)** RNA expression. **(G)** Spearman’s correlation analysis of GBP1 and ERα in CHB patients treated with peg IFNα-2b. **(H)** ROC curve analysis for evaluating the sensitivity and specificity of the GBP1 and ERα levels to predict the response to peg IFNα-2b treatment in patients with CHB. **(I)** Immunohistochemical staining for ERα and GBP1 in nonresponder and responder liver tissues. All the data are presented as the means ± SDs; *P < 0.05, ****P < 0.0001.

To evaluate whether ERα and GBP1 are linked to the HBV infection process and interferon efficacy, peripheral blood samples from 66 adult CHB patients treated with peg IFNα-2b and 17 healthy controls were collected. This cohort comprised 40 male cases (60.6%) and 26 female cases (39.4%). Patients treated with Peg-IFNα-2b were categorized into a treatment group (n = 66) and a functional cure group (n = 34). Peripheral blood was collected from the treatment group before treatment, after 3 and 6 months of treatment, and from the functional cure group for testing. Moreover, liver biopsy tissue was obtained from six patients. Immunohistochemical staining was used to examine the expression of ERα and GBP1. To clarify whether sex-related factors confound the core study outcomes, we performed a balance analysis of core indicators and key baseline characteristics across sex subgroups within both response and nonresponse cohorts. The evaluated parameters included: baseline serum GBP1 and ERα expression levels before treatment, pretreatment serum HBsAg load, and dynamic changes in GBP1 expression during the treatment course. The results demonstrated that none of the above baseline or core indicators differed significantly between male and female participants (all P > 0.05; detailed data are presented in **Supplementary Table S1**). As shown in **Table 2**, **Figure 9A**, GBP1 levels were significantly higher in patients with a functional cure and those with pretreatment, 3 months, and 6 months posttreatment compared to healthy controls (*P* < 0.0001). However, the treatment group and the functional cure group did not differ (*P* > 0.05). Patients with CHB had marginally lower levels of GBP1 mR, significantly lower than those in healthy controls, before treatment. However, with peg IFN-α-2b treatment, GBP1 levels increased (*P* < 0.05) (**Figure 9B**). Additionally, ERα mRNA expression in CHB patients did not significantly change during treatment. However, it showed a downward trend compared with that of the clinical cure group and the healthy control group (**Figure 9C**).

**Table 2:** Clinical characteristics of the patients.

Groups	Parameters				
	Health control	CHB (Untreated)	CHB (Treated for 3 months)	CHB (Treated for 6 months)	CHB (Clinical cure)
Cases	17	66	66	66	34
Age (years)	25.82 ± 3.78	41.39 ± 9.33	41.39 ± 9.33	41.39 ± 9.33	41.39 ± 9.33
Sex (M/F)	9/8	40/26	40/26	40/26	22/12
Serum HBsAg level (IU/mL)	NA	1325.7 (115.1925, 4980.29)	591.925 (27.3575, 2136.5275)	191.63 (3.24, 1883.2025)	LDL
HBV DNA level (log10 IU/mL)	NA	LDL	LDL	LDL	LDL
ALT (U/L)	20.42 ± 5.33	25.50 (15.75, 40.50)	46.50 (33.00, 40.25)	34.50 (19.00, 50.50)	39.00 (19.75, 55.00)
AST (U/L)	18.29 ± 5.73	23.00 (18.00, 32.00)	39.00 (31.75, 66.50)	33.00 (21.75, 47.00)	26.5 (20.75, 45.75)
WBC (*10^9/ml)	3.55 ± 0.85	5.76 ± 1.29	3.43 ± 1.26	4.23 ± 2.43	5.14 ± 1.37
Serum GBP1 level (ng/mL)	1.28 ± 0.20	1.90 ± 0.304	1.92 ± 0.35	1.94 ± 0.38	1.95 ± 0.32
GBP1 RNA	1.93 (0.95, 2.19)	1.66 (1.08, 2.87)	4.34 (2.80, 10.00)	4.30 (3.05, 6.39)	3.46 (2.47, 5.74)
ERα RNA	1.16 (0.85, 1.99)	0.93 (0.37, 1.82)	0.94 (0.38, 1.50)	0.64 (0.22, 1.56)	1.59 (0.67, 2.25)
TB (μmol/L)	8.87 ± 2.05	12.48 ± 5.96	10.80 ± 4.19	10.02 ± 3.84	10.73 ± 3.50
DB (μmol/L)	4.86 ± 1.34	4.58 ± 1.59	5.01 ± 1.69	4.47 ± 1.44	4.49 ± 1.61

ALT, alanine aminotransferase; AST, aspartate aminotransferase; F, female; M, male; HBsAg, HBV surface antigen; WBC, white blood cell; TB, total bilirubin; DB, direct bilirubin; HC, healthy control; NA, not applicable; LDL, low detection limit.

We then compared the clinical characteristics of patients in the treatment group at 3 and 6 months post-treatment (**Table 3**). According to the treatment recommendations for chronic hepatitis B, a total of 66 patients were categorized into two groups based on a rapid decline in HBsAg (HBsAg <200 IU/mL or a decline of>1 log10 IU/mL at 12 or 24 weeks), which served as the early response criterion. Our results revealed that 40 patients had an early response, and 26 were nonresponders. GBP1 expression did not differ significantly between the two groups; however, the response group exhibited greater levels of ERα and GBP1 than the nonresponse group (**Figures 9D-F**). Next, we observed a correlation between ERα and GBP1 via Spearman’s correlation analysis. Our results suggested a noteworthy association between the level of ERα mRNA in patients before treatment and the level of GBP1 mRNA during treatment (r = 0.87, 0.83, 0.84; all *P* < 0.0001). Furthermore, the positive correlation between ERα mRNA and GBP1 mRNA was found to be even greater in patients who achieved a clinical cure (r = 0.74, *P* < 0.0001) (**Figure 9G**). To investigate the value of GBP1 and ERα expression in assessing the early response to peg IFNα-2b treatment, we plotted ROC curves for pretreatment, 3 months of treatment, and 6 months of treatment using GBP1 and ERα as reference values and obtained areas under the curve (AUCs) of 0.583, 0.668, and 0.862, respectively. GBP1 and ERα demonstrated statistically significant but moderately predictive accuracy for the pretreatment with IFNα-2b. We found that the levels of ERα and GBP1 after 6 months of treatment had the highest predictive value for early response to peg IFNα-2b treatment (**Figure 9H**).

**Table 3:** Clinical characteristics of patients with different effects.

Groups	Parameters					
	Nonresponse			Response		
	Untreated	Treated for 3 months	Treated for 6 months	Untreated	Treated for 3 months	Treated for 6 months
Cases	26			40		
Age (years)	41.23 ± 9.74			41.50 ± 9.20		
Sex (M/F)	20/6			20/20		
Serum HBsAg level (IU/mL)	2609.66 (1207.33, 6220.14)	1808.84 (717.47, 3382.60)	1865.20 (892.63, 3423.27)	460.85 (52.63, 2827.56)	65.20 (4.90, 1030.97)	9.30 (0.26, 193.35)
ALT (U/L)	29.50 (21.75, 39.00)	49.50 (33.75, 71.00)	32.50 (18.75, 65.50)	20.00 (15.00, 41.50)	44.50 (29.25, 70.00)	36.50 (19.50, 47.75)
AST (U/L)	22.5 (18.50, 28.75)	39.50 (29.75, 59.25)	33.50 (19.00, 52.00)	23.00 (18.00,32.00)	38.00 (32.00, 69.50)	32.5 (22.00, 46.25)
WBC (*10^9/ml)	5.92 ± 1.31	3.35 ± 1.27	4.11 ± 1.56	5.64 ± 1.28	3.48 ± 1.26	4.30 ± 2.79
TB (μmol/L)	13.13 ± 6.63	11.04 ± 3.95	10.77 ± 4.25	10.94 (7.78, 14.95)	10.65 ± 4.38	9.52 ± 3.48
DB (μmol/L)	4.80 ± 1.71	4.98 ± 1.45	4.50 ± 1.49	4.43 ± 1.50	5.04 ± 1.85	4.44 ± 1.40
Serum GBP1 level (ng/mL)	1.93 ± 0.29	1.79 ± 0.33	1.76 ± 0.37	1.88 ± 0.32	2.00 ± 0.34	2.06 ± 0.34
GBP1 RNA	1.46 (1.11, 1.84)	3.28 (1.14, 4.89)	3.38 (2.42, 4.58)	1.97 (1.10, 3.04)	5.42 (3.66,11.98)	5.09 (3.77, 10.83)
ERα RNA	0.71 (0.34, 2.19)	0.67 (0.14, 1.90)	0.44 (0.17, 1.80)	0.97 (0.57, 1.61)	0.98 (0.76, 1.38)	0.85 (0.22, 1.48)

ALT, alanine aminotransferase; AST, aspartate aminotransferase; F, female; M, male; HBsAg, HBV surface antigen; WBC, white blood cell; TB, total bilirubin; DB, direct bilirubin.

To further analyze the correlation between ERα and GBP1, we collected liver tissue samples from 6 patients treated with peg IFNα-2b, of which 3 had a good response and achieved a clinical cure, and 3 responded poorly. ERα was predominantly expressed in the nucleus, whereas GBP1 was more obviously expressed in the cytoplasm, validating the results of previous cellular experiments. Moreover, compared with patients in the nonresponsive group, patients in the responsive group presented increased expression of ERα and GBP1 in liver tissue (**Figure 9I**).

### 3.10 GBP1 is correlated with innate immunity to IFN-α

**Figure 10:**
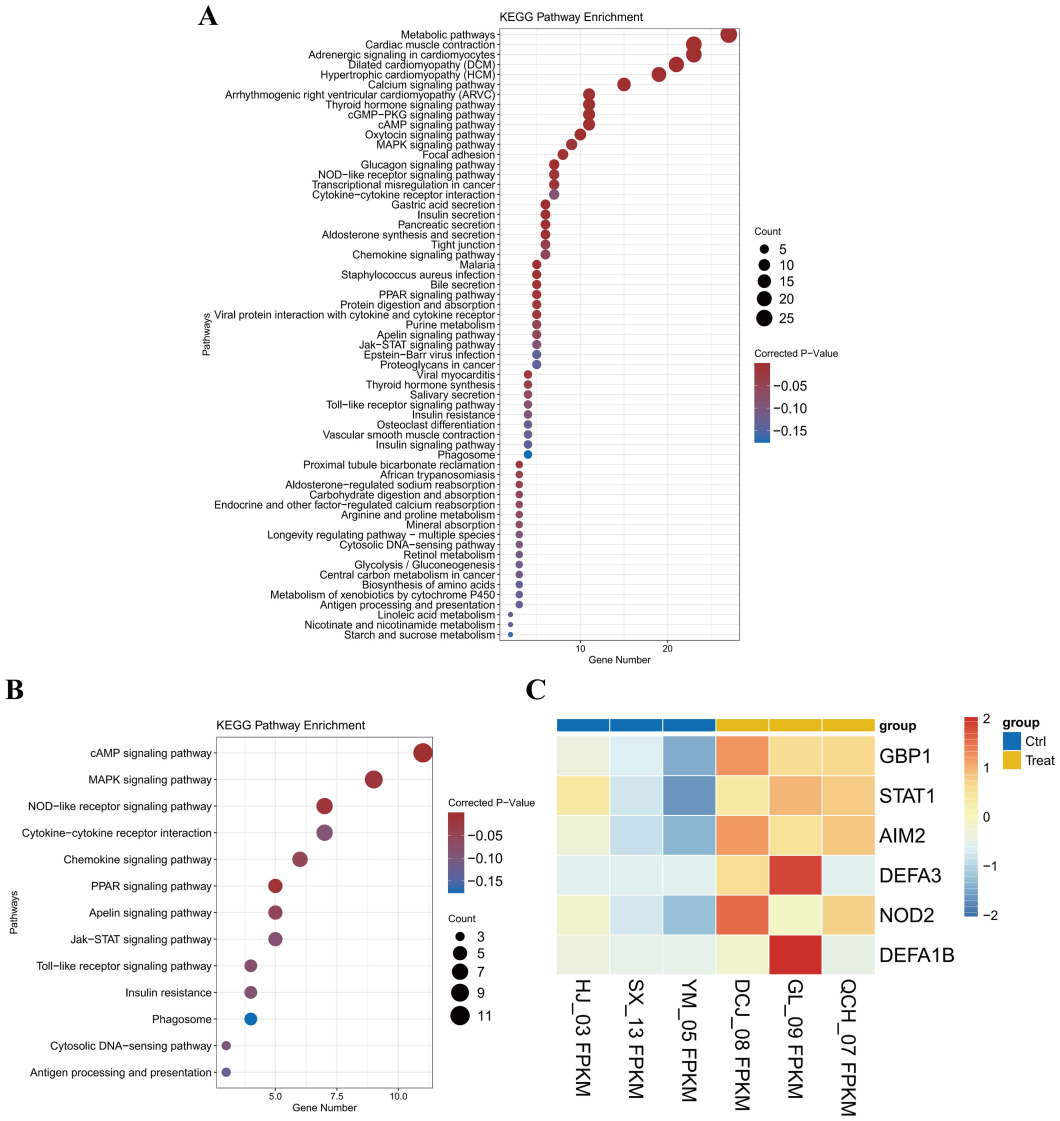
GBP1 was correlated with innate immunity to IFN-α. **(A)** KEGG pathway enrichment of RNA sequences in the liver tissues of 6 CHB patients. **(B)** KEGG pathway enrichment of the top 13 innate immune signalling pathways associated with the RNA sequence in the liver tissues of 6 CHB patients. **(C)** GBP1 was enriched in the NOD-like signalling pathway.

We subsequently compared differentially expressed genes (DEGs) between good responders and poor responders through RNA-seq. The results indicated that GBP1 was among the differentially expressed genes, whereas ERα was not significantly different between the two groups. An analysis of the KEGG pathways revealed that the DEGs were highly enriched in immune-related pathways (**Figures 10A, B**). Further analysis revealed that GBP1 was enriched in the NOD-like signalling pathway (**Figure 10C**), which activates the host immune response and is closely associated with inflammasome activation, the production of the caspase family, and pyroptosis (41–43). These data provide valuable information about the immune activation of peg IFNα-2b and therapeutic approaches for CHB in future studies. Furthermore, these findings suggest that the activation of innate immune mechanisms plays a vital role in achieving a clinical cure for CHB.

## 4 Discussion

In recent years, multiple clinical studies have confirmed that the use of IFN-α alone or in conjunction with NAs can effectively enhance HBsAg clearance, achieve a functional cure, and significantly reduce the occurrence and progression of end-stage liver disease (44–46). To manage the hepatitis B virus, current guidelines recommend IFN-α as the drug of choice. According to clinical studies, young women exhibit a greater propensity for early response to interferon therapy and a shorter duration to achieve clinical remission than men do (22, 27). It has been suggested that the sex difference in interferon therapy is due to the influence of female sex hormone levels. A variety of biological pathways are regulated by estrogen signalling via ERα and Erβ (40). ERα is the main receptor for estrogen in hepatocytes and is involved in processes such as hepatocyte proliferation and apoptosis, providing a protective effect on hepatocytes. The regulatory effect of ERα on HBV is reflected in many aspects, such as HBV invasion, transcription, replication, and the development of HBV-associated HCC (47, 48). ERα not only regulates the Th1/Th2 balance of an immune response but can also affect the activity of NTCP receptors and inhibit HBV infection (49). When ERα enters the nucleus of infected hepatocytes, it can compete with HBV enhancer element I (EnhI) to bind the transcription factor HNF4α and inhibit HBV transcription and replication (23). Although the mechanisms of ERα and IFN-α against HBV have been widely explored, their combined mechanism of action has rarely been reported. In this study, we investigated the combined mechanism of ERα and IFN-α, identifying the response effector GBP1. GBP1, a member of the GBP family, is associated with innate immunity and antiviral function (49). GBP1 is one of the early interferon-induced ISGs and plays a crucial role in inhibiting HBV replication (50, 51). Our previous study on the effect of GBP1 on HBV replication revealed that GBP1 can inhibit HBV replication and promote HBsAg clearance (33). In this study, we further explored and discovered that ERα suppresses HBV transcription and replication by targeting and promoting the expression of GBP1, thereby enhancing the patient response to IFN-α treatment. Combining clinical observations and bioinformatics analyses,.We found that GBP1 was upregulated in individuals who responded well to interferon treatment, and the differential expression of GBP1 was consistent with the dysregulation of interferon-related pathways identified through bioinformatics analysis. This indicates that the abnormal expression of GBP1 is associated with the general disorder of the interferon signaling pathway.

GBP1 is a member of the GBP family and is associated with innate immunity and antiviral functions (50). GBP1 is one of the early interferon-induced ISGs and plays a crucial role in inhibiting HBV replication (51, 52). In this study, we demonstrated that GBP1 acted as an intersection gene between ERα and peg IFNα-2b. ERα combined with peg IFNα-2b inhibited HBV transcription and replication by increasing the expression of the intersection gene GBP1. We subsequently clarified the precise mechanism by which ERα and peg IFNα-2b inhibit HBV. Notably, our data demonstrated the interaction between ERα and GBP1, leading to the suppression of HBV replication. Based on the observation that HBV promotes GBP1 expression, we hypothesized that IFN-α and ERα therapy could disrupt the equilibrium between GBP1 and HBV by stimulating GBP1 expression, thereby facilitating the elimination of HBV. This finding supports the role of IFN-α and ERα in eliminating HBV through immune-related pathways. It is necessary to study further the effect of GBP1 on the anti-HBV activity of peg IFNα-2b and ERα. In our current research, we found that ERα and peg IFNα-2b induced GBP1 expression and reduced the expression of total HBV RNA, 3.5 kb RNA, HBsAg, HBeAg, HBV core protein, and HBV surface protein. In addition, we found that silencing GBP1 promoted HBsAg and HBeAg production, and increased the levels of total HBV RNA, 3.5 kb RNA, and HBsAg protein, whereas overexpressing GBP1 increased the level of GBP1 in the context of viral infection. Surprisingly, cotreatment with GBP1 and peg IFNα-2b further increased the antiviral effect of IFN, whereas cotreatment with GBP1 silencing and peg IFNα-2b partly restored the inhibitory effect of GBP1 on HBV in Huh7-ERα cells. Therefore, GBP1 can effectively modulate peg IFNα-2b and ERα, thus suppressing HBV transcription and replication.

Molecular dynamics simulation and molecular docking once again proved that the binding of ERα and GBP1 was stable. In our present study, we found that the binding site of ERα and GBP1 was concentrated in the EF region, which is involved in sensitivity to estrogen, receptor dimerization, nuclear localization, and critical roles for coactivators or coinhibitors (53, 54). In this study, we confirmed that the EF region is a key binding site for ERα and GBP1 via qRT-PCR and Western blotting. Importantly, ERα overexpression markedly increased GBP1 expression in the nucleus, whereas loss of the EF region significantly diminished the nuclear expression of GBP1 and impaired the inhibition of HBs proteins. Interestingly, we found that ERα was predominantly expressed in the nucleus by immunofluorescence, a finding consistent with other studies (55). Therefore, we believe that ERα overexpression may increase the protein expression level of GBP1 within the nucleus, which is performed mainly through the EF region of ERα.

Given the significant involvement of GBP1 and ERα in HBV infection, we investigated the correlation between GBP1 and ERα and the effectiveness of peg IFNα-2b therapy in individuals diagnosed with CHB. We found that the serum levels of GBP1 in CHB patients were higher compared to those in healthy controls, and that the expression of GBP1 mRNA increased more significantly after PEG IFNα-2b treatment. Furthermore, patients with CHB who have a higher GBP1 level might respond better to peg IFNα-2b. It is imperative to conduct additional research on the expression levels of ERα and GBP1 in pregnant IFNα-2b clinical responders or nonresponders. Despite the absence of any notable disparity in ERα expression across all groups, further observation revealed a marginal increase in ERα expression within the response group compared with the nonresponse group. Spearman correlation analyses revealed a substantial positive correlation between ERα and GBP1 expression in both the treatment group and the functional cure group prior to treatment. ROC curves verified the potential value of GBP1 and ERα in predicting interferon efficacy. Although the prediction accuracy is limited, it has certain statistical significance. In the future, larger clinical samples are needed for cautious verification. Combined with the functional mechanism we elucidated, it indicates that the ERα-GBP1 axis may be of significance in the clinical course of hepatitis B virus infection. Therefore, we suggest that the interaction between ERα and GBP1 has a positive effect on interferon treatment of CHB and may contribute to the improvement of HBsAg clearance. The immunohistochemical results further confirmed the increased level of GBP1 expression in CHB patients with a good response to peg IFNα-2b treatment. Moreover, we discovered that GBP1 was enriched in the NOD-like signalling pathway, which activates the host immune response (56–58). Therefore, we believe that the possible focus of HBV clearance *in vivo* remains on the remodeling and restoration of host immune mechanisms.

In summary, this study revealed that ERα and IFNα can stimulate the expression of GBP1. It also reveals that the ERα-GBP1 axis is a novel node in the HBV-host interaction network.The combination of ERα and peg IFNα-2b therapy *in vitro* demonstrated an antiviral effect by facilitating elevated GBP1 expression. Moreover, our clinical study demonstrated that GBP1 can be used to predict the clinical response to IFNα-2b in CHB patients. These discoveries could elucidate the resistance mechanism of peg IFN-2b and aid in the design of therapeutic medications for patients who do not respond to peg IFN-2b. However, it remains unclear how GBP1 interacts with known HBV proteins and other host factors to jointly shape the viral replication environment. Hepatitis B virus (HBV) pathogenesis relies on intricate reticular interactions between viral effector proteins and host cellular proteins (59), forming a complex regulatory network governing disease progression. Our study identifies GBP1 as a differential gene between HBV patients with distinct treatment responses, verifying its interaction with ERα, subcellular localization dynamics, and serum secretion. As an IFN-responsive gene, GBP1 may integrate into this network via IFN signaling crosstalk or direct/indirect interactions with HBV proteins (e.g., HBcAg, HBx), potentially modulating viral replication or host transcriptional programs. Future studies using TAP-MS, CRISPR/Cas9, or multi-omics approaches to dissect GBP1’s role will deepen understanding of HBV pathogenesis and resistance mechanisms, offering novel insights for system biology-based targeted therapies to improve outcomes, especially for poor responders (60). It should be emphasized that this study analyzed the expression of GBP1 at a single time point. Given the dynamic nature of the liver transcriptome during the progression of liver diseases, the expression level of GBP1 may vary at different stages of chronic hepatitis B infection. Future longitudinal cohort studies that depict the expression trajectory of GBP1 throughout the disease course will be of great value and provide new targets for developing new combination treatment strategies (59).

## Data Availability

The datasets presented in this study can be found in online repositories. The names of the repository/repositories and accession number(s) can be found in the article/**Supplementary Material**.
